# Dehydroepiandrosterone sulfate (DEHA-S), cortisol and adrenocorticotropic hormone (ACTH) levels in drug-naive, first episode patients with psychosis

**DOI:** 10.1192/j.eurpsy.2021.2137

**Published:** 2021-08-13

**Authors:** P. Petrikis, S. Tigas, M. Christou, S. Brikos, A. Karampas, G. Georgiou, T. Hyphantis

**Affiliations:** 1 Department Of Psychiatry, UNIVERSITY OF IOANNINA-FACULTY OF MEDICINE, IOANNINA, Greece; 2 Department Of Endocrinology, UNIVERSITY OF IOANNINA-FACULTY OF MEDICINE, IOANNINA, Greece

**Keywords:** DEHA-S, ACTH, psychosis, cortisol

## Abstract

**Introduction:**

Impaired response to stress and a pathological activation of the hypothalamic-pituitary-adrenal axis have been implicated in the pathophysiology of schizophrenia

**Objectives:**

To measure serum ACTH, cortisol and DEHA-S levels in drug-naïve, first-episode patients with psychosis.

**Methods:**

Results are reported as mean (standard deviation, range). Paired t-test or Wilcoxon signed rank test were performed for group comparisons. The level of significance was set at p-value<0.05. Statistical analysis was performed with Stata 15.1.

**Results:**

Data were included for 110 subjects (70 men, 40 women); 55 patients and 55 controls matched for age and sex. Mean age was 31.3 years (8.7, 18-48) in patients and 31.4 years (8.9, 17-49) in controls. Serum cortisol and Cortisol/DHEA-S ratio were statistically significantly lower in patients [12.6μg/dl (4.5, 3.5-24.5) and 5.3 (3.6, 1.3-19.5), respectively] compared to controls [15.5 μg/dl (4.9, 4.2-30.1) and 8 (4.7, 1.1-25.5), respectively] (p-value=0.0068 and 0.0005, respectively). Additionally, serum DHEA-S was statistically significantly higher in patients [306.5 μg/dl(165.4, 70-790)] compared to controls [240.1 μg/dl(113.5, 46-597)] (p-value=0.0114). ACTH was also higher in patients [28.5 pg/ml(15.7, 6.2-73.9)] than controls [26.5 pg/ml (15.3, 7-70.5)] but this difference wasn’t statistically significant (p-value=0.6359).

**Conclusions:**

We report elevated DEHA-S, decreased cortisol levels and decreased cortisol/DEHA-S ratio in the patients’ compared to the controls’ group.

**Disclosure:**

No significant relationships.

